# Effects of disability on income and income composition

**DOI:** 10.1371/journal.pone.0286462

**Published:** 2023-05-31

**Authors:** Christy Pu, Huei-Fen Syu

**Affiliations:** Institute of Public Health, National Yang Ming Chiao Tung University, Taipei, Taiwan; University of Milano–Bicocca: Universita degli Studi di Milano-Bicocca, ITALY

## Abstract

**Background:**

Income composition is highly associated with individual financial sustainability and income inequality at the macro level. Although studies have investigated the effects of disability on wage income, few studies have investigated the effects of disability on income composition or on various types of income other than wage income.

**Methods:**

We sampled 72,000 households using tax data sourced from the Taiwan Ministry of Finance in 2015. Data for each household member were traced back to 1999. We identified 23,346 individuals with disabilities and matched them with 34,145 individuals without disabilities. Eight income types were identified. A two-way fixed-effect analysis was performed to determine the effects of disability on changes in each income type. Fractional probit models were estimated to determine the effects of disability on the proportion of each income type in total income at different ages.

**Results:**

Wage income constitutes the largest proportion of income in Taiwan. The total income is estimated to increase by 10.4% (P < 0.001) after disability onset. Moreover, most income categories did not experience a decline following the onset of disability. We also noted a significant interaction effect between disability status and age on the proportion of each income type in total income.

**Conclusion:**

The effect of disability on income varied across different sources of income. The income composition observed for the individuals with disabilities changed considerably at various ages. Accordingly, policies should be designed to ensure long-term sustainability of income sources for individuals with disabilities.

## Introduction

According to the World Health Organization (WHO) statistics in 2021, more than 1 billion people live with some form of disability, accounting for approximately 15% of the global population, and up to 190 million people aged 15 years and older have functional difficulties [1]. Individuals with disabilities may be particularly disadvantaged financially because they are likely to incur high medical expenses. Disability is associated with poverty measures [2]. According to the WHO, individuals with disabilities are 50% more likely to incur catastrophic health spending than their nondisabled counterparts [1], making personal financial sustainability an essential topic for improving quality of life for individuals with disabilities.

Studies have mainly investigated how disability affects labor wages [3–8], and health policy studies have often overlooked different sources of income for individuals with disabilities. Individuals with disabilities are increasingly excluded from the labor market [9], and the onset of a work-preventing disability is associated with a quick decline in earnings [10]. Disability may reduce hourly productivity, the ability to work long hours, and the labor participation rate [3,4,11]. Reduced labor productivity due to ill health accounts for a wage gap of up to 35% between individuals with disabilities and those without [4]. Discrimination against individuals with disabilities is also salient and contributes to the wage differential [3].

Wage differentials between individuals with and without disabilities can vary significantly with age [3]. The literature exhibits a gap, namely a lack of research on how disability affects sources of income other than labor earnings and whether this relationship is affected by age. Age is a variable that should be investigated because it can be a key determinant of income level and type. For example, older adults may be eligible for pensions and social transfers; these factors can lead to differences in income level and structure between age groups. In addition, age-related factors (e.g., pension programs) are substantially associated with various forms of income inequality [12,13]; thus, age is a key factor in the analysis of policy design.

A study suggested that even if the wage gap were narrowed, the employment gap would still remain because in addition to productivity loss, a high proportion of unexplained variance exists in the disability–wage equation [3]. Accordingly, researchers must investigate the question as to whether individuals with disabilities compensate for the reduction in wages by seeking other sources of income that are not influenced by employment discrimination or labor productivity loss. For example, earnings from stock dividends or savings interest are less likely to be affected by discrimination and labor productivity.

Income composition is a major indicator of income inequality [14]. Paul [15] reported that business income, wages, salaries, and property income tend to increase income inequality, whereas government cash benefits, pensions, and other regular income sources can reduce inequality. Other scholars have noted the effects of different income types on income inequality [16,17]. This demonstrates that rather than merely investigating whether disability engenders a decrease in wages, studies should investigate changes in the sources of incomes for individuals with disabilities. A study conducted in Finland discovered that income sources vary significantly for people with rejected disability pension applications [18]; this finding indicates that people can adjust their income sources after their disability pension applications are rejected on the basis of their disability status. Whether people with severe disabilities who qualify for government welfare or tax exemptions change their income sources upon obtaining disability benefits is unclear.

Another limitation of existing studies investigating the association between disability and earnings is that they have generally been based on self-reported disability status and/or income [2,3,6,8,10]. However, substantial measurement errors and nonresponses can occur in surveys of income, especially in surveys querying sources of income and income levels [19]. Moreover, nonworkers appear to systematically overreport disability status in surveys [20].^.^ Such reporting bias can lead to biased conclusions regarding the true effects of disability on earnings.

To address the aforementioned limitations, the present study investigated the effects of disability on different sources of earnings by analyzing administrative tax data. In Taiwan, individuals with disabilities receive an additional tax allowance and are entitled to welfare benefits. Individuals receiving such benefits are enrolled in the Disability Registry, rendering tax data a reliable source for identifying disability status and different income sources.

## Methods

### Data

The Taiwan Ministry of Finance (MOF) manages publicly available tax data and distributes said data to researchers upon request by using block sampling. Specifically, the data are stratified by income and randomly sampled. When a person was selected through sampling, tax-related information for all household members was also visible. A total of 72,000 households were identified using the 2015 tax data. We then traced the data for each household member backward until 1999. Notably, data samples are generally drawn by the MOF for research purposes and do not contain all households in Taiwan. However, the samples have been verified by the MOF to be representative of the population. More details can be found on the MOF’s official website [21]. This study was approved by the Institutional Review Board (IRB) of National Yang Ming Chiao Tung University (approval number: YM110069E). Written informed consents from individuals were waived by the above named IRB.

The collected data contained abundant information on financial status and disability status. They also contained information on detailed income composition. “Household income” was the basis for income tax declaration; however, earnings were recorded on an individual basis. Several verification data points such as individual and household income, different categories of deductions, and tax withholding were included. The tax data did not include information on households with income below the minimum taxable amount.

In Taiwan, disability applications must be completed by the applicant. Upon application, the applicant must indicate the disability type. The applicant is then assessed by a physician from a designated medical institution. In this context, disability is not solely defined on the basis of activities of daily living (ADLs). Instead, physicians are required to exercise their professional judgement, and disability status is approved and granted after an evaluation in which a physician assesses and rate disability severity. Information on severity is unavailable in the database used in the present study. Because a person with registered disability status enjoys public welfare benefits such as tax allowances, employment assistance, tax exemption, and certain medical allowances, those qualified for disability status are likely to register. Nevertheless, we could not identify disability types or durations in the tax data used in this study because tax allowances do not vary by these factors.

### Income and other variables

Within the collected tax file, each individual’s income was categorized into the following types: (1) income from profit-seeking; (2) income from professional practice; (3) income from salaries and wages; (4) interest income; (5) income from leases and income from royalties; (6) income from self-undertaking in farming, fishing, animal husbandry, forestry, and mining; (7) income from property transactions; (8) income from contests, games, prizes, and awards won by chance; (9) severance pay or retirement pay; and (10) other income. During the study period, only a few individuals had income from categories (6) and (10); therefore, we analyzed only the remaining eight categories. Detailed definitions for each type of income are provided in Table 1. Residential area was identified from the dataset and was divided into the following categories: North-Central, Central, South-Central, South, and Others.

**Table 1:** Income types and definitions.

Income types	Definitions
Profit-seeking	Dividends from company stocks, profits from partnerships or proprietorships, and personal commercial profits
Professional practice	Income from practice as lawyer, accountant, architect, technician, physician, pharmacist, midwife, writer, agent, land administrator, artisan, and other skilled professional, deducting cost of practice
Salary and wage	Wages, salaries, bonuses, and allowances from employers
Interest	Interest from bonds and savings
Lease and royalty	Rental income from property and income from land ownership and down payment, deducting costs such as depreciation
Self-undertaking in farming, fishing, animal husbandry, forestry, and mining	Income from self-undertaking in farming, fishing, animal husbandry, forestry, and mining, deducting operating costs
Property transactions	Income from property transactions, deducting original cost of obtaining property and cost of other investment on the property
Contests, games, prizes, and awards won by chance	Income from contests, games, prizes, and awards won by chance
Severance pay or retirement pay	Pension, severance pay, lifelong stipend, annuity
Other income	Other income not specified, such as employee stock subscription and specific types of employee benefits

### Statistical methods

In this study, we considered individuals as the unit of analysis. We investigated the effect of disability on various types of income through linear regression by using a two-way fixed-effects model. For this purpose, we generated a new variable that indicated the year in which the individual became disabled. This variable was thus time variant. In addition, we included a year variable in our model to assess the effect of time. For this part of the analysis, only individuals with disabilities were included because those without disabilities would not have had a time-varying disability status variable.

A log of zero is undefined; therefore, we replaced income with the value “1” for individuals with zero income. We included a set of control variables, namely age, age squared (a variable for capturing the nonlinear effect of age on income), and place of residence.

To assess whether the proportion of each income type in total income changes by disability status, we matched each individual with a disability with two individuals without disabilities according to age and sex where possible. We did not use residential area as a matching variable because of the limited number of people available for matching; instead, we controlled for this variable in our regression analysis. To ensure feasible matching, we conducted the age matching by using 5-year blocks for people aged between 20 and 65 years. Individuals aged younger than 20 years were not analyzed for comparability reasons. Individuals aged older than 65 years were categorized into one age group. We matched individuals by ensuring a balanced age and sex distribution across all years of data. For each individual with disabilities, the year of the first disability record in the database was identified as the index year. For individuals without disabilities, the index year was defined as the index year of the matched individuals with disabilities.

We estimated *fractional probit* models to obtain quasi-likelihood estimators. We defined total income as the sum of the eight types of income, and we calculated the proportion of each income type in total income. Disability was entered as a dummy variable. In this model, variables are used similarly to how they were used in the aforementioned linear model. We further added an interaction term between disability status and age. Subsequently, we calculated the marginal effect of disability on the proportion of each income type in total income. Moreover, we derived conditional means for the proportion of each income type in total income stratified by disability status. All statistical analyses were performed using STATA 15 [22].

## Results

We matched each individual with disabilities with two individuals without disabilities according to age and sex where possible. Thus, we matched a total of 34,145 individuals without disabilities (one of the study groups) with 23,346 individuals with disabilities (the other study group). Table 2 presents the sample characteristics, indicating the distribution of age, sex, and area of residence. Because sex was perfectly matched, we observed no significant difference in sex between the two groups. Age and residential area remained unbalanced; hence, we controlled for these variables in all subsequent regression analyses.

**Table 2:** Sample characteristics.

	Disabled (*n* = 23,346	)	Nondisabled (*n* = 34,145)		*P* value
	*n*	Mean/%	*n*	Mean/%	
Age		59.7		51.1	<0.001
SD		(18.1)		(17.7)	
min		(20)		(20)	
max		(107)		(105)	
Sex (male)	12,600	53.97	17,472	51.17	1.000
Place					
North	6455	27.65	9468	27.73	<0.001
North-central	3302	14.14	5003	14.65	
Central	4487	19.22	6720	19.68	
South-central	4494	19.25	6190	18.13	
South	2982	12.77	4643	13.6	
Others	1626	6.96	2121	6.21	

**Fig 1:**
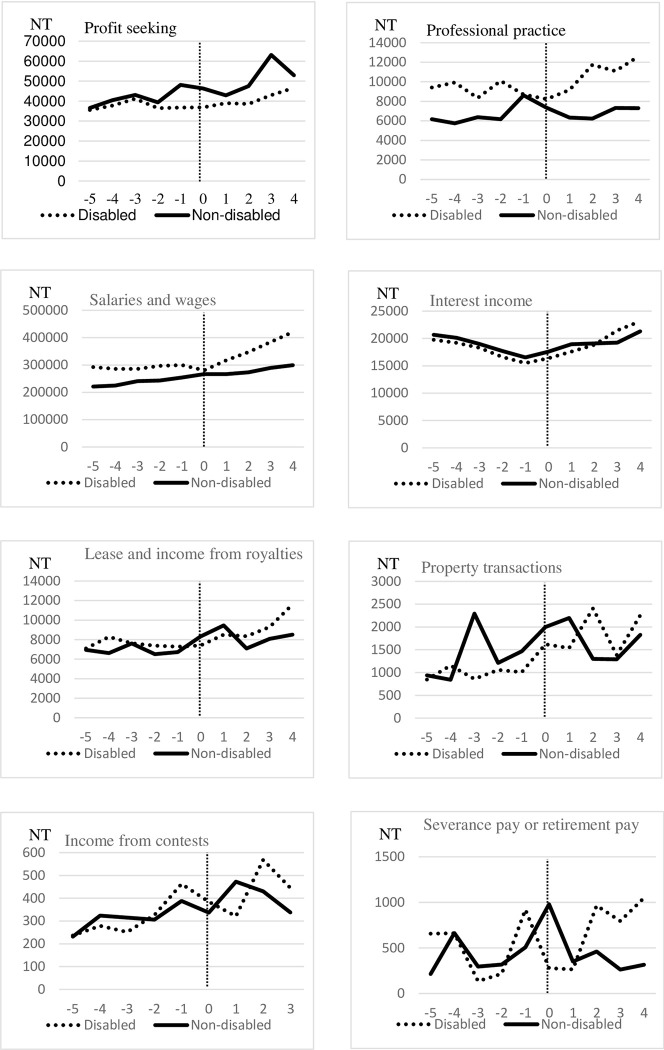
Various income types between individuals with and without disabilities. Fig 1 illustrates a comparison of the various income types between individuals with disabilities and those without disabilities at various time points. The index date was defined as *Time* = 0. Exchange rate: US$1 is approximately NT$30.9 (March 2023).

Fig 1 illustrates a comparison of the various income types between individuals with disabilities and those without disabilities at various time points. The index date was defined as *Time* = 0. Before the index date, individuals with disabilities had lower profit-seeking, interest, and property transaction income than did those without disabilities; however, they had higher income from professional practice, salary and wage, and lease and royalty income. Notably, our data sample included individuals with income above the taxable threshold; this may thus explain why the individuals with disabilities had higher income in certain categories prior to the index date.

Table 3 presents the results of two-way fixed-effects estimation for multiple income types. The results revealed that the total income increased by 10.4% (P < 0.001) after the onset of disability. Moreover, no decrease in wage income was observed; in fact, most income categories exhibited an increase.

**Table 3:** Estimation for the relationship between disability and various types of income.

	Estimates	Robust standard error	95% CI		*P* value
Total	0.104	0.021	0.062	0.146	<0.001
Profit-seeking	0.113	0.020	0.073	0.153	<0.001
Professional practice	0.040	0.016	0.009	0.071	0.012
Salaries and wages	0.153	0.025	0.104	0.202	0.000
Interest	0.063	0.023	0.017	0.108	0.007
Leases and royalties	0.005	0.012	-0.018	0.028	0.657
Property transactions	0.024	0.014	-0.003	0.050	0.079
Contests	0.034	0.016	0.003	0.065	0.031
Severance pay or retirement pay	-0.00013	0.005	-0.009	0.009	0.979

CI: Confidence interval.

To determine whether the proportion of each income type in total income would change, we further estimated fractional probit models. Table 4 lists the unadjusted proportions of each income type in total income for both groups before and after the index date.

**Table 4:** Proportion of each type of income in total income.

	Before index date					After index date				
	Disabled		Nondisabled		*P* value	Disabled		Nondisabled		*P* value
Income type	Mean	SD	Mean	SD		Mean	SD	Mean	SD	
Profit-seeking	0.153	0.309	0.164	0.320	<0.001	0.162	0.318	0.168	0.327	0.0015
Professional practice	0.020	0.121	0.017	0.110	<0.001	0.024	0.133	0.018	0.117	<0.001
Salaries and wages	0.571	0.457	0.555	0.461	<0.001	0.569	0.460	0.572	0.463	0.3679
Interest	0.229	0.385	0.232	0.385	0.049	0.213	0.372	0.209	0.371	0.1073
Leases and royalties	0.020	0.115	0.024	0.127	<0.001	0.000	0.000	0.025	0.132	0.184
Property transactions	0.004	0.053	0.004	0.053	0.784	0.005	0.056	0.004	0.051	0.007
Contests	0.003	0.044	0.003	0.050	0.004	0.003	0.048	0.004	0.053	0.1766
Severance pay or retirement pay	0.000	0.014	0.001	0.017	0.058	0.000	0.013	0.000	0.013	0.4938

Wage and salary income constitute the largest proportion of total income for both groups before and after the index date. However, before the index date, the proportion of wage and salary income in total income was significantly higher for individuals with disabilities (*P* < 0.001); by contrast, after the index date, this proportion was higher for individuals without disabilities *(P =* 0.368). Interest and profit-seeking income constituted the second- and third-largest proportions of total income, respectively, for both groups. The proportion of profit-seeking income was slightly lower in the individuals with disabilities both before (*P* < 0.001) and after (*P* = 0.002) the index date.

**Fig 2:**
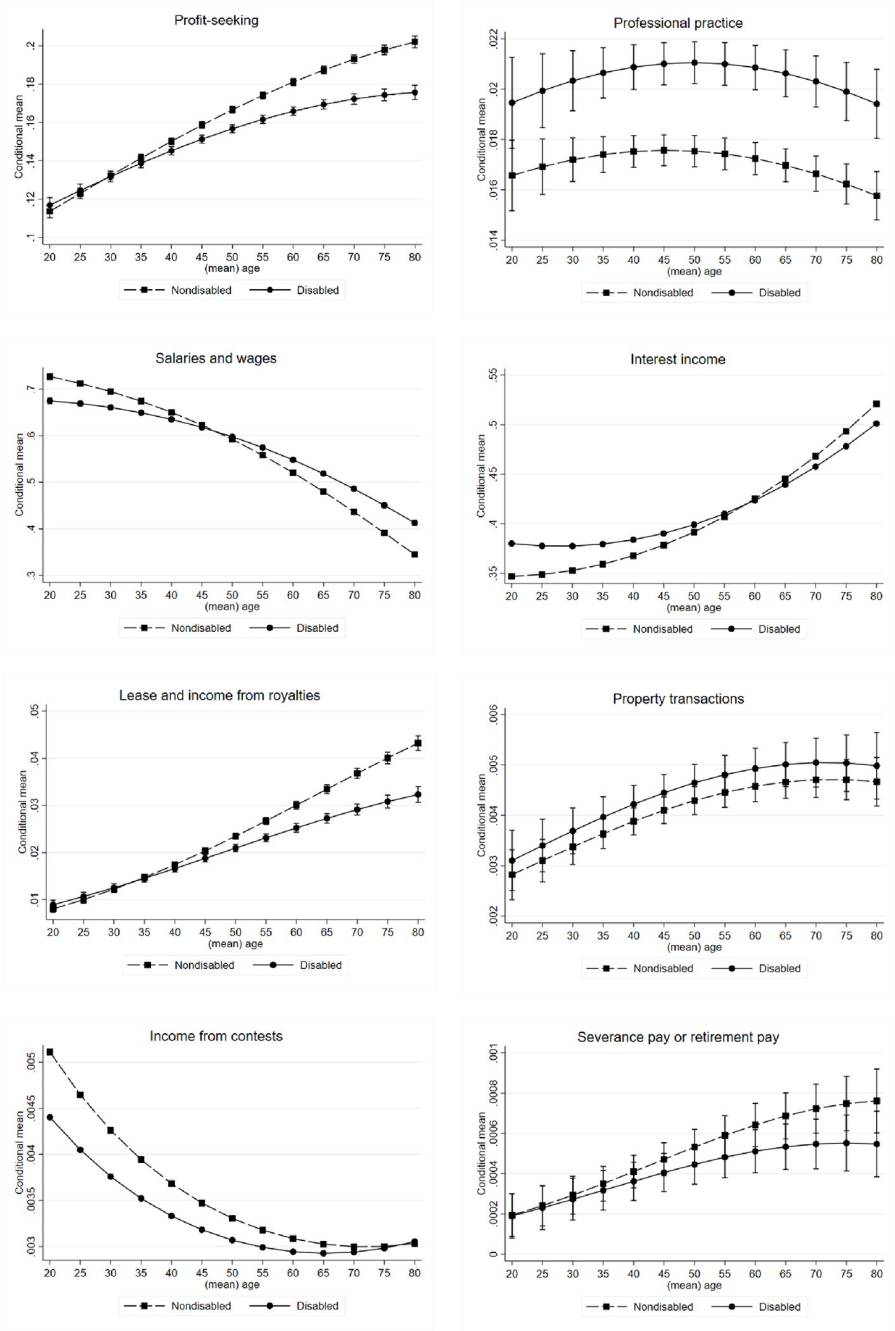
Proportions of the different income types at various ages. Fig 2 displays the conditional means derived for the proportions of the different income types at various ages, estimated using fractional probit models. The number represent predicted proportion of each income type in total income.

Fig 2 displays the conditional means derived for the proportions of the different income types at various ages. The effects of age on the conditional means derived for the proportions of the different income types varied considerably. The conditional means derived for the proportions of profit-seeking, interest, lease and royalty, property transaction, and severance pay or retirement pay income increased with age.

The conditional means derived for the proportion of interest income exhibited an exponential relationship with age and indicated the existence of a significant interaction between disability and age. The predicted proportion of interest income in total income was higher in the individuals with disabilities than in those without disabilities before the age of 50 years. However, a reverse trend was observed after the age of 50 years; that is, the proportion of interest income in total income was higher in the individuals without disabilities. For individuals with disabilities aged 75–80 years, interest income constituted approximately 35% of total income, and for individuals with disabilities aged 20–25 years, interest income constituted only approximately 16% of total income.

The conditional means derived for the proportion of profit-seeking income also demonstrated a significant interaction between disability and age. The difference in the predicted proportion increased after the age of 35 years. Specifically, for individuals with disabilities aged 75–80 years, profit-seeking income constituted approximately 18% of total income, and for individuals with disabilities aged 20–25 years, profit-seeking income constituted only approximately 12% of total income. This finding was expected because this income type primarily originates from dividends from company stock investments, and older individuals are more likely to have such investments in their asset portfolios.

The conditional means derived for the proportion of wage and salary income decreased as age increased and indicated a significant interaction between disability and age. For individuals with disabilities aged 20–25 years, wage and salary income constituted approximately 68% of total income. We observed a slightly higher proportion for individuals without disabilities. The proportion of wage and salary income decreased continually as age increased.

## Discussion

Income for individuals with disabilities is directly related to quality of life and is thus an essential topic in health policymaking. The present study investigated the effects of disability on changes in income from various sources. The study derived four main findings: (1) Contrary to our hypothesis, upon disability onset, income did not decrease. (2) Wage and salary income constituted the largest proportion of total income for individuals with and without disabilities. (3) The proportions of the various income types in total income varied considerably with age, regardless of disability status. (4) The proportions of the various income types in total income were significantly affected by the interaction between disability status and age.

This study has several merits. First, we used administrative data, which prevented reporting bias. Second, we included several income sources to analyze changes in the proportions of income types rather than merely demonstrating effects on wage and salary income. From a methodological perspective, the use of panel data enabled us to eliminate time-invariant fixed effects.

In the event of health shocks with unanticipated expenses, households may increase their borrowing, labor supply may be adjusted, and assets may be depleted [23]. An individual may earn income from different sources, but not all sources of income have equal effects on the individual’s spending patterns. For example, Sand [24] revealed that people with different types of income have different marginal propensities to consume. In another study, Kirt et al. discovered that only certain types of household income affect children’s growth patterns [25]. Accordingly, examining changes in income from different sources can provide valuable insight into how financial status is adjusted after a disability.

In the fractional probit models, income composition varied substantially by age. In Taiwan, individuals with disabilities who are aged older than 65 years account for 44.4% of all individuals with disabilities; individuals with disabilities aged 50–65 years and those aged older than 65 years accounted for up to 72.2% of all individuals with disabilities in 2019 [26]. Because the disabled population mainly consists of older adults, the analysis of income composition by age is crucial for policy formulation.

Costa [27] found that the earnings of individuals who apply for Social Security Disability Insurance benefits declined rapidly in the years prior to application, and older applicants experienced longer decline periods prior to application than did younger applicants. Additionally, older individuals with disabilities are more likely to be unable to work full-time compared with younger counterparts [28]. Rather than remain in a disadvantageous working environment over the long term, newly disabled older adults are likely to retire or move to a less demanding job compared with younger ones.

In our fractional probit analysis, the predicted results indicated that relative to people without disabilities, those with disabilities derived a higher fraction of their total income from wage earnings. This does not imply that the absolute wages (measured in dollar amounts) of people with disabilities are higher than those of those without disabilities; instead, proportion is the key point of discussion in this context. A study revealed that economically disadvantaged individuals tend to derive a higher proportion of their income from labor income, whereas wealthy individuals tend to derive their income equally from both labor income and capital income; this difference is a key contributor to income inequality [14]. Accordingly, policies should be designed to protect individuals with disabilities in the labor market, especially older adults with disabilities. Specifically, policies should allow individuals with disabilities to remain in the labor market and retain high-paying jobs at older age. Moreover, older adults with disabilities excessively rely on labor income as a key contributor to total income, and this overreliance engenders income inequality, which should be a policy concern.

The analysis using the two-way fixed-effects model revealed no decrease in wage income after disability onset. Time-invariant factors that are unmeasured or unobserved can affect the wage earnings of an individual. Gannon and Munley investigated wage earnings and found that the factors explaining the wage differentials between individuals with and without disabilities varied significantly with age; more unexplained wage gaps were observed between individuals with and without disabilities aged 45–65 years, and the wage gap between individuals with and without disabilities decreased over time for these older individuals [3]. These unobserved factor can be correlated with both risk of become disabled and income. For example, people with higher wage may be working in a more hazardous environment, or have harsher working conditions that prevent them from having a healthier life, and these environment and working conditions are likely to be correlated with higher risk of being disabled, despite they may get higher pay. Meyier and Mok [29] found a huge decline in income ten years after disability onset. We did not find a significant reduction in wage income upon obtaining disability status in our sample. One explanation is that we did not investigate the long-term effect of disability on wage income, instead, we analyzed how income change in the short term. People with disability may decide to retire in the long run and thus reduced their income. Another explanation is that in Taiwan, welfare policies for people with disability differ from that of other country. For example, Taiwan has National Health Insurance that significantly enabled increased healthcare utilization for people with disability compared with people without disability [30]. This may partially mitigate the negative health effect on wage income for people with disability.

We found that the individuals with disabilities had increased interest income after disability onset compared with individuals without disabilities. A possible explanation for this finding is that private disability insurance schemes provided compensation for the disability onset, leading to higher savings and hence interest income. Another possible explanation is that the individuals’ households sold assets and properties to raise cash to meet anticipated future expenses associated with the disability, thereby resulting in increased savings in the short term. This also explains why we observed an increase in income from property transactions approximately 2 years after the index date. However, policymakers should note that this type of increased income relies on one-time payouts and is not likely to be persistent; thus, individuals with disabilities may face financial instability in the long term if they rely on increased earnings from interest.

The time variable in our analysis exerted a significant effect on multiple types of income. Unobservable factors that change over time may exist. At individual level, an individual’s risk preferences may be time variant and correlated with both the risk of becoming disabled and income levels [31]. Such risk preferences can vary owing to broader macroeconomic factors over time. Productivity is another potential time-varying factor.

We observed that the proportions of income types varied considerably among different age groups, and certain income types were significantly affected by the interaction effect between disability status and age. For older adults, profit-seeking income tended to constitute a large proportion of their total income; profit-seeking income was primarily composed of earnings from stock dividends. This finding is consistent with those of Charles and Kasilingam [32], who reported that age is one of the key determinants of investment behavior; our finding is also consistent with that of Bergantino [33], who revealed that the scale and composition of household assets change considerably with the age of household heads. Furthermore, our study demonstrated that for older individuals, interest income constituted a smaller proportion of the total income of those with disabilities compared with that of those without disabilities. This may be because older individuals with poorer health are more likely to have less savings compared with their healthier counterparts [34]^.^ At older age, assets may deplete faster for individuals with disabilities.

How disability was measured in different studies should be considered when comparing results. In our study, disability was measured on the basis of the disability status that qualifies an individual for a tax exemption, and this status is assessed and determined by a physician. Other studies have used other measures such as self-reported information regarding limitations in terms of daily or functional activities [2,3]. Thus, the application of our measure could have resulted in a sample consisting of a high proportion of people with severe disabilities.

This study has some limitations, which should be considered when interpreting its findings. First, as discussed previously, only households that met the minimum threshold required to pay income tax were included in this study. These households thus represent relatively wealthy households in the country. Therefore, the results should not be generalized to the entire country; individuals with lower incomes may have alternative sources of income that may decrease upon the onset of disability. Second, the tax data did not provide many variables for use in our regression as controls. However, we used two-way fixed-effects models; therefore, our results should remain robust as long as these variables are time invariant. Third, because disability type does not affect the tax allowance that is granted, the data did not contain this variable; therefore, we could not distinguish between types of disabilities in our analysis.

## Conclusion

Disability status imparts different effects on different types of income. The effects of disability on income composition were noted to vary with age. Policies should be designed to ensure the long-term sustainability of income sources for individuals with disabilities.

## Supporting information

S1 Appendix (DOCX)

## References

[1] World Health Orginization. Disability and health. https://wwwwhoint/news-room/fact-sheets/detail/disability-and-health Accessed June 1, 2022. 2021.

[2] Brucker DL, Mitra S, Chaitoo N, Mauro J. More likely to be poor whatever the measure: Working‐age persons with disabilities in the United States. Social Science Quarterly. 2015;96(1):273–96. 10.1111/ssqu.12098 PMC13227786 42239490

[3] Gannon B, Munley M. Age and disability: explaining the wage differential. Social Science & Medicine. 2009;69(1):47–55. doi: 10.1016/j.socscimed.2009.04.013 19464783

[4] Madden D. Labour market discrimination on the basis of health: an application to UK data. Applied Economics. 2004;36(5):421–42.

[5] Malo MÁ, Pagán R. Wage differentials and disability across Europe: Discrimination and/or lower productivity? International Labour Review. 2012;151(1‐2):43–60.

[6] Mitra S, Sambamoorthi U. Wage differential by disability status in an agrarian labour market in India. Applied Economics Letters. 2009;16(14):1393–8.

[7] Baldwin ML, Choe C. Re-examining the models used to estimate disability-related wage discrimination. Applied Economics. 2014;46(12):1393–408.

[8] Kidd MP, Sloane PJ, Ferko I. Disability and the labour market: an analysis of British males. Journal of health economics. 2000;19(6):961–81. doi: 10.1016/s0167-6296(00)00043-6 11186853

[9] Hao Y, Xiao R. How Disability Income Benefits Affect Employment for Persons with Disabilities in China: An Impairment-Based Work Disability Assessment Perspective. Int J Environ Res Public Health. 2022;19(6). Epub 20220314. doi: 10.3390/ijerph19063428 ; PubMed Central PMCID: PMC8951574. 35329116 PMC8951574

[10] Singleton P. Insult to injury disability, earnings, and divorce. Journal of Human Resources. 2012;47(4):972–90.

[11] Schultz TP, Tansel A. Wage and labor supply effects of illness in Cote d’Ivoire and Ghana: instrumental variable estimates for days disabled. Journal of development economics. 1997;53(2):251–86.

[12] Brown RL, Prus SG. Social transfers and income inequality in old age: A multinational perspective. North American Actuarial Journal. 2004;8(4):30–6.

[13] Karunaratne HD. Age as a factor determining income inequality in Sri Lanka. The Developing Economies. 2000;38(2):211–42.

[14] Ranaldi M. Income composition inequality. Review of Income and Wealth. 2022;68(1):139–60.

[15] Paul S. Income sources effects on inequality. Journal of Development Economics. 2004;73(1):435–51.

[16] Lerman RI, Yitzhaki S. Income inequality effects by income source: A new approach and applications to the United States. The review of economics and statistics. 1985:151–6.

[17] Silber J. Inequality decomposition by income source: a note. The Review of Economics and Statistics. 1993:545–7.

[18] Perhoniemi R, Blomgren J, Laaksonen M. Sources of income following a rejected disability pension application: a sequence analysis study. Disability and Rehabilitation. 2020;42(15):2161–9. doi: 10.1080/09638288.2018.1555619 31081397

[19] Moore JC, Stinson LL, Welniak EJ. Income measurement error in surveys: A review. Journal of Official Statistics-Stockholm-. 2000;16(4):331–62.

[20] Kreider B, Pepper JV. Disability and employment: Reevaluating the evidence in light of reporting errors. Journal of the American Statistical Association. 2007;102(478):432–41.

[21] Taiwan Ministry of Finance. https://wwwfiagovtw/singlehtml/19ce7984c7734d7dba479a954c88530f Accessed on 30 May, 2022.

[22] StataCorp. 2017. Stata Statistical Software: Release 15. College Station, TX: StataCorp LLC.

[23] Mohanan M. Causal effects of health shocks on consumption and debt: quasi-experimental evidence from bus accident injuries. Review of Economics and Statistics. 2013;95(2):673–81. doi: 10.1162/REST_a_00262 28003706 PMC5166431

[24] Sand R. The propensity to consume income from different sources and implications for saving: an application to Norwegian farm households. 2002.

[25] Kirk A, Kilic T, Carletto C. Composition of household income and child nutrition outcomes evidence from Uganda. World Development. 2018;109:452–69. doi: 10.1016/j.worlddev.2017.03.023 30177866 PMC6018066

[26] Ministry of Health, Taiwan. https://depmohwgovtw/dos/cp-5224-62359-113html Accessed on 30 April 2022.

[27] Costa J. The Decline in Earning Prior to Application for Disability Insurance Benefits. Soc Sec Bull. 2017;77:1.

[28] Boersema HJ, Hoekstra T, Abma F, Brouwer S. Inability to Work Fulltime, Prevalence and Associated Factors Among Applicants for Work Disability Benefit. J Occup Rehabil. 2021;31(4):796–806. Epub 20210312. doi: 10.1007/s10926-021-09966-7 ; PubMed Central PMCID: PMC8558289. 33710457 PMC8558289

[29] Meyer BD, Mok WK. Disability, earnings, income and consumption. Journal of Public Economics. 2019;171:51–69.

[30] Hou C, Pu C. Association between visual impairment and health care utilization. American Journal of Ophthalmology. 2021. 10.1016/j.ajo.2021.07.033 34407430

[31] Anderson LR, Mellor JM. Predicting health behaviors with an experimental measure of risk preference. Journal of health economics. 2008;27(5):1260–74. doi: 10.1016/j.jhealeco.2008.05.011 18621427

[32] Charles MA, Kasilingam DR. Does the investor’s age influence their investment behaviour? Paradigm. 2013;17(1–2):11–24.

[33] Bergantino SM. Life cycle investment behavior, demographics and asset prices: Massachusetts Institute of Technology; 1998.

[34] De Nardi M, French E, Jones JB. Life expectancy and old age savings. American Economic Review. 2009;99(2):110–15.

