# Supplementary material for: Effects of disability on income and income composition
Source: PLoS One. 2023 May 31;18(5):e0286462. doi: 10.1371/journal.pone.0286462 (PMC10231772; doi:10.1371/journal.pone.0286462)
Supplement: S1 Appendix — (DOCX) [file pone.0286462.s001.docx]

Appendix A: Full estimation for the relationship between disability and income (fixed effect models)

| Total income | |  |  |  |  |
| --- | --- | --- | --- | --- | --- |
|  | Estimates | Standard error | 95 % confidence interval | | P value |
| Disability (yes=1) | 0.104 | 0.021 | 0.062 | 0.146 | <0.001 |
| Age | 0.015 | 0.002 | 0.010 | 0.020 | <0.001 |
| Age^2 | -0.0001426 | 0.0000219 | -0.000186 | -0.0000996 | <0.001 |
| Place of residence (Reference=North) | | | |  |  |
| North-central | -0.055 | 0.044 | -0.141 | 0.031 | 0.213 |
| Central | -0.196 | 0.046 | -0.286 | -0.106 | <0.001 |
| South-central | -0.287 | 0.045 | -0.375 | -0.200 | <0.001 |
| South | -0.386 | 0.051 | -0.486 | -0.287 | <0.001 |
| Others | -0.386 | 0.063 | -0.510 | -0.262 | <0.001 |
| Year (Reference=2009) | | |  |  |  |
| 2010 | 0.029 | 0.020 | -0.009 | 0.068 | 0.136 |
| 2011 | 0.184 | 0.023 | 0.139 | 0.230 | <0.001 |
| 2012 | 0.233 | 0.027 | 0.180 | 0.286 | <0.001 |
| 2013 | 0.261 | 0.030 | 0.202 | 0.320 | <0.001 |
| 2014 | 0.381 | 0.033 | 0.316 | 0.446 | <0.001 |
| 2015 | 0.406 | 0.035 | 0.337 | 0.476 | <0.001 |
| Constant | 8.765 | 0.070 | 8.628 | 8.901 | <0.001 |
|  |  |  |  |  |  |
| Income from profit-seeking | | |  |  |  |
|  | Estimates | Standard error | 95 % confidence interval | | P value |
| Disability (yes=1) | 0.113 | 0.020 | 0.073 | 0.153 | <0.001 |
| Age | 0.006 | 0.002 | 0.002 | 0.010 | 0.003 |
| Age^2 | -0.000059 | 0.000017 | -0.0000923 | -0.0000258 | <0.001 |
| Place of residence (Reference=North) | | | |  |  |
| North-central | -0.124 | 0.042 | -0.207 | -0.042 | 0.003 |
| Central | -0.222 | 0.042 | -0.304 | -0.139 | <0.001 |
| South-central | -0.198 | 0.040 | -0.276 | -0.119 | <0.001 |
| South | -0.334 | 0.047 | -0.426 | -0.243 | <0.001 |
| Others | -0.339 | 0.055 | -0.446 | -0.231 | <0.001 |
| Year (Reference=2009) | | |  |  |  |
| 2010 | 0.355 | 0.020 | 0.315 | 0.395 | <0.001 |
| 2011 | 0.538 | 0.024 | 0.492 | 0.584 | <0.001 |
| 2012 | 0.382 | 0.026 | 0.330 | 0.433 | <0.001 |
| 2013 | 0.359 | 0.029 | 0.302 | 0.416 | <0.001 |
| 2014 | 0.451 | 0.032 | 0.388 | 0.514 | <0.001 |
| 2015 | 0.525 | 0.035 | 0.456 | 0.593 | <0.001 |
| Constant | 3.383 | 0.056 | 3.273 | 3.493 | <0.001 |
|  |  |  |  |  |  |
| Income from professional practice | | |  |  |  |
|  | Estimates | Standard error | 95 % confidence interval | | P value |
| Disability (yes=1) | 0.040 | 0.016 | 0.009 | 0.071 | 0.012 |
| Age | 0.004 | 0.001 | 0.001 | 0.007 | 0.011 |
| Age^2 | -0.0000372 | 0.0000129 | -0.0000624 | -0.000012 | 0.004 |
| Place of residence (Reference=North) | | | |  |  |
| North-central | -0.078 | 0.033 | -0.143 | -0.012 | 0.020 |
| Central | -0.082 | 0.031 | -0.142 | -0.022 | 0.007 |
| South-central | -0.102 | 0.030 | -0.160 | -0.044 | 0.001 |
| South | -0.124 | 0.038 | -0.199 | -0.049 | 0.001 |
| Others | -0.142 | 0.041 | -0.222 | -0.062 | 0.001 |
| Year (Reference=2009) | | |  |  |  |
| 2010 | -0.009 | 0.015 | -0.039 | 0.020 | 0.546 |
| 2011 | -0.002 | 0.017 | -0.035 | 0.032 | 0.914 |
| 2012 | -0.028 | 0.019 | -0.065 | 0.008 | 0.129 |
| 2013 | 0.006 | 0.021 | -0.035 | 0.047 | 0.780 |
| 2014 | 0.034 | 0.023 | -0.012 | 0.080 | 0.153 |
| 2015 | -0.007 | 0.026 | -0.058 | 0.044 | 0.788 |
| Constant | 0.585 | 0.044 | 0.499 | 0.671 | <0.001 |
|  |  |  |  |  |  |
| Income from salaries and wages | | |  |  |  |
|  | Estimates | Standard error | 95 % confidence interval | | P value |
| Disability (yes=1) | 0.153 | 0.025 | 0.104 | 0.202 | <0.001 |
| Age | 0.010 | 0.003 | 0.005 | 0.016 | <0.001 |
| Age^2 | -0.0000883 | 0.0000244 | -0.0001362 | -0.0000404 | <0.001 |
| Place of residence (Reference=North) | | | |  |  |
| North-central | -0.124 | 0.051 | -0.224 | -0.025 | 0.014 |
| Central | -0.218 | 0.053 | -0.322 | -0.115 | <0.001 |
| South-central | -0.323 | 0.052 | -0.425 | -0.221 | <0.001 |
| South | -0.471 | 0.062 | -0.593 | -0.350 | <0.001 |
| Others | -0.449 | 0.073 | -0.592 | -0.305 | <0.001 |
| Year (Reference=2009) | | |  |  |  |
| 2010 | 0.071 | 0.021 | 0.029 | 0.113 | 0.001 |
| 2011 | 0.069 | 0.026 | 0.017 | 0.120 | 0.009 |
| 2012 | 0.168 | 0.031 | 0.107 | 0.229 | <0.001 |
| 2013 | 0.039 | 0.035 | -0.030 | 0.108 | 0.271 |
| 2014 | 0.054 | 0.040 | -0.024 | 0.132 | 0.173 |
| 2015 | 0.006 | 0.043 | -0.080 | 0.091 | 0.899 |
| Constant | 6.113 | 0.080 | 5.956 | 6.270 | <0.001 |
|  |  |  |  |  |  |
|  |  |  |  |  |  |
| Interest income | |  |  |  |  |
|  | Estimates | Standard error | 95 % confidence interval | | P value |
| Disability (yes=1) | 0.063 | 0.023 | 0.017 | 0.108 | 0.007 |
| Age | 0.007 | 0.002 | 0.003 | 0.012 | 0.002 |
| Age^2 | -0.0000754 | 0.0000206 | -0.0001157 | -0.0000351 | <0.001 |
| Place of residence (Reference=North) | | | |  |  |
| North-central | -0.008 | 0.047 | -0.100 | 0.083 | 0.860 |
| Central | -0.085 | 0.047 | -0.178 | 0.008 | 0.074 |
| South-central | -0.219 | 0.044 | -0.306 | -0.132 | <0.001 |
| South | -0.191 | 0.051 | -0.291 | -0.092 | <0.001 |
| Others | -0.303 | 0.060 | -0.420 | -0.186 | <0.001 |
| Year (Reference=2009) | | |  |  |  |
| 2010 | -0.283 | 0.022 | -0.326 | -0.241 | <0.001 |
| 2011 | -0.057 | 0.026 | -0.108 | -0.006 | 0.028 |
| 2012 | 0.109 | 0.029 | 0.052 | 0.167 | <0.001 |
| 2013 | 0.247 | 0.033 | 0.182 | 0.311 | <0.001 |
| 2014 | 0.386 | 0.037 | 0.314 | 0.458 | <0.001 |
| 2015 | 0.493 | 0.040 | 0.415 | 0.570 | <0.001 |
| Constant | 3.992 | 0.065 | 3.864 | 4.120 | <0.001 |
|  |  |  |  |  |  |
|  |  |  |  |  |  |
| Income from leases and income from royalties | | | |  |  |
|  | Estimates | Standard error | 95 % confidence interval | | P value |
| Disability (yes=1) | 0.005 | 0.012 | -0.018 | 0.028 | 0.657 |
| Age | 0.001 | 0.001 | -0.001 | 0.003 | 0.309 |
| Age^2 | -0.0000105 | 0.0000997 | -0.0000301 | 0.000095 | 0.292 |
| Place of residence (Reference=North) | | | |  |  |
| North-central | 0.013 | 0.027 | -0.041 | 0.067 | 0.638 |
| Central | 0.013 | 0.026 | -0.038 | 0.064 | 0.611 |
| South-central | 0.010 | 0.021 | -0.032 | 0.051 | 0.647 |
| South | -0.017 | 0.025 | -0.066 | 0.033 | 0.514 |
| Others | -0.047 | 0.029 | -0.104 | 0.010 | 0.104 |
| Year (Reference=2009) | | |  |  |  |
| 2010 | 0.027 | 0.011 | 0.005 | 0.048 | 0.015 |
| 2011 | 0.012 | 0.013 | -0.014 | 0.038 | 0.378 |
| 2012 | 0.025 | 0.016 | -0.006 | 0.055 | 0.111 |
| 2013 | 0.053 | 0.017 | 0.019 | 0.087 | 0.002 |
| 2014 | 0.061 | 0.019 | 0.024 | 0.098 | 0.001 |
| 2015 | 0.076 | 0.021 | 0.036 | 0.117 | <0.001 |
| Constant | 0.511 | 0.033 | 0.447 | 0.575 | <0.001 |
|  |  |  |  |  |  |
| Income from property transactions | | |  |  |  |
|  | Estimates | Standard error | 95 % confidence interval | | P value |
| Disability (yes=1) | 0.024 | 0.014 | -0.003 | 0.050 | 0.079 |
| Age | 0.001 | 0.001 | -0.002 | 0.003 | 0.492 |
| Age^2 | -0.0000922 | 0.0000112 | -0.0000312 | 0.0000128 | 0.411 |
| Place of residence (Reference=North) | | | |  |  |
| North-central | -0.026 | 0.027 | -0.080 | 0.027 | 0.330 |
| Central | -0.029 | 0.027 | -0.083 | 0.024 | 0.280 |
| South-central | -0.075 | 0.024 | -0.121 | -0.028 | 0.002 |
| South | -0.095 | 0.027 | -0.148 | -0.041 | 0.001 |
| Others | -0.051 | 0.033 | -0.114 | 0.013 | 0.120 |
| Year (Reference=2009) | | |  |  |  |
| 2010 | -0.017 | 0.015 | -0.046 | 0.011 | 0.237 |
| 2011 | -0.035 | 0.015 | -0.065 | -0.005 | 0.024 |
| 2012 | -0.041 | 0.016 | -0.072 | -0.009 | 0.011 |
| 2013 | -0.022 | 0.017 | -0.055 | 0.012 | 0.206 |
| 2014 | -0.054 | 0.019 | -0.091 | -0.018 | 0.004 |
| 2015 | -0.080 | 0.020 | -0.120 | -0.040 | <0.001 |
| Constant | 0.189 | 0.035 | 0.120 | 0.258 | <0.001 |
|  |  |  |  |  |  |
| Income from contests, games, prizes, and awards won by chance | | | | |  |
|  | Estimates | Standard error | 95 % confidence interval | | P value |
| Disability (yes=1) | 0.034 | 0.016 | 0.003 | 0.065 | 0.031 |
| Age | 0.003 | 0.002 | -0.0001774 | 0.006 | 0.065 |
| Age^2 | -0.0000277 | 0.0000132 | -0.0000536 | -0.0000174 | 0.036 |
| Place of residence (Reference=North) | | | |  |  |
| North-central | -0.002 | 0.033 | -0.067 | 0.063 | 0.956 |
| Central | -0.021 | 0.029 | -0.078 | 0.036 | 0.471 |
| South-central | -0.033 | 0.030 | -0.092 | 0.026 | 0.268 |
| South | -0.075 | 0.034 | -0.141 | -0.008 | 0.028 |
| Others | -0.120 | 0.039 | -0.197 | -0.044 | 0.002 |
| Year (Reference=2009) | | |  |  |  |
| 2010 | 0.080 | 0.016 | 0.049 | 0.111 | <0.001 |
| 2011 | 0.116 | 0.017 | 0.083 | 0.150 | <0.001 |
| 2012 | 0.085 | 0.018 | 0.050 | 0.121 | <0.001 |
| 2013 | 0.113 | 0.020 | 0.074 | 0.153 | <0.001 |
| 2014 | 0.138 | 0.022 | 0.095 | 0.182 | <0.001 |
| 2015 | 0.152 | 0.025 | 0.104 | 0.200 | <0.001 |
| Constant | 0.258 | 0.043 | 0.172 | 0.343 | <0.001 |
|  |  |  |  |  |  |
| Severance pay or retirement pay | | |  |  |  |
|  | Estimates | Standard error | 95 % confidence interval | | P value |
| Disability (yes=1) | -0.000127 | 0.005 | -0.009 | 0.009 | 0.979 |
| Age | -0.00016630 | 0.001 | -0.001 | 0.001 | 0.762 |
| Age^2 | 0.00000466 | 0.0000506 | -0.000094 | 0.0000104 | 0.927 |
| Place of residence (Reference=North) | | | |  |  |
| North-central | -0.003 | 0.011 | -0.025 | 0.018 | 0.754 |
| Central | 0.0004358 | 0.011 | -0.022 | 0.023 | 0.970 |
| South-central | -0.001 | 0.011 | -0.022 | 0.021 | 0.948 |
| South | -0.016 | 0.018 | -0.050 | 0.019 | 0.373 |
| Others | -0.009 | 0.014 | -0.037 | 0.018 | 0.505 |
| Year (Reference=2009) | | |  |  |  |
| 2010 | -0.002 | 0.005 | -0.011 | 0.007 | 0.648 |
| 2011 | 0.001 | 0.005 | -0.009 | 0.011 | 0.779 |
| 2012 | -0.003 | 0.005 | -0.014 | 0.007 | 0.522 |
| 2013 | 0.002 | 0.006 | -0.010 | 0.014 | 0.769 |
| 2014 | 0.005 | 0.007 | -0.008 | 0.018 | 0.453 |
| 2015 | 0.007 | 0.008 | -0.008 | 0.022 | 0.336 |
| Constant | 0.026 | 0.015 | -0.003 | 0.056 | 0.083 |
